# Analysis of hemolysis-associated acute myeloid leukemia genes obtained using weighted gene co-expression network analysis and a Mendelian randomization study

**DOI:** 10.1007/s44313-025-00073-7

**Published:** 2025-04-11

**Authors:** Rui Zhang, Yan Zang, Linguo Wan, Hui Yu, Zhanshan Cha, Haihui Gu

**Affiliations:** Department of Transfusion Medicine, the, First Affiliated Hospitalaq , Naval Military Medical University, Shanghai, 200433 China

**Keywords:** Hemolysis, AML, Weighted gene co-expression network analysis, Mendelian randomization, Toll-like receptor 4 (TLR4)

## Abstract

**Purpose:**

We used bioinformatics methods and Mendelian randomization (MR) analysis to investigate the hub genes involved in acute myeloid leukemia (AML) and their causal relationship with hemolysis, to explore a new direction for molecular biology research of AML.

**Methods:**

We first differentially analyzed peripheral blood samples from 62 healthy volunteers and 65 patients with AML from the Gene Expression Omnibus database to obtain differentially expressed genes (DEGs), and intersected them with genes sourced from weighted gene co-expression network analysis (WGCNA) and the GeneCards database to obtain target genes. Target genes were screened using protein–protein interaction (PPI) network analysis and ROC curves to identify genes associated with AML. Finally, we analyzed the correlation between genes and immune cells and the relationship between toll-like receptor 4 (TLR4) and AML using MR.

**Results:**

We compared peripheral blood expression profiles using an array of 62 healthy volunteers (GSE164191) and 65 patients with AML (GSE89565) (M0:25; M1:11; M2:10; M3:1; M4:7; M4 eo t [16;16] ou inv [16]:4; M5:6; M6:1) and obtained 7,339 DEGs (3,733 upregulated and 3,606 downregulated). We intersected these DEGs with 4,724 genes from WGCNA and 1,330 genes related to hemolysis that were identified in the GeneCards database to obtain 190 target genes. After further screening these genes using the PPI network, we identified TLR4, PTPRC, FCGR3B, STAT1, and APOE, which are closely associated with hemolysis in patients with AML. Finally, we found a causal relationship between TLR4 and AML occurrence using MR analysis (*p* < 0.05).

**Conclusion:**

We constructed a WGCNA-based co-expression network and identified hemolysis-associated AML genes.

**Supplementary Information:**

The online version contains supplementary material available at 10.1007/s44313-025-00073-7.

## Introduction

Acute myeloid leukemia (AML) is preceded by malignant clonal expansion of progenitor cells with impaired differentiation and is associated with the highest proportion of deaths among all types of leukemia [1–4]. In the United States, in 2024, there were 20,800 new cases of AML, accounting for 33.1% of the total incidence of leukemia, the 9 th and 10 th most prevalent malignancies in men and women, respectively, and 11,220 deaths, accounting for 47.4% of the total mortality rate of leukemia, ranking as the 6 th most common death due to cancer in men and the 8 th in women [5]. AML can lead to life-threatening infections, bleeding, hemolysis, and anemia [6]. Therefore, blood product inputs are clinically recommended to reverse anemia, control hemolysis, and reduce bleeding [6], thereby improving patient survival.

Although there is a strong relationship between blood product input and hemolysis [7, 8], the causes of hemolysis are more complex in patients with AML [9, 10]. Patients with leukemia develop thrombotic microangiopathy, which can cause intravascular hemolysis [11]. The therapeutic disposition to leukemia can also lead to hemolysis, possibly because of immune reconstitution and interferon release after a patient receives a bone marrow transplant [12]. There have also been case reports that gemtuzumab ozogamicin (Mylotarg™), a therapeutic agent for AML, may cause intravascular hemolysis in patients with low or normal blood potassium concentrations [13]. This shows that hemolysis is closely related to the prognosis of patients with AML; however, the reasons for the occurrence of hemolysis in patients with AML are not fully understood.

Existing research has focused on how to avoid hemolysis and whether new AML therapies cause hemolysis [14–17]. Moreover, determining the expression levels of specific genes is important for understanding the mechanisms of hemolysis in patients with AML, as well as for prognostic assessment. In this study, we used bioinformatics methods, such as weighted gene co-expression network analysis (WGCNA), to identify crucial genes involved in the hemolytic process in patients with AML and Mendelian randomization (MR) to infer causal relationships between exposure factors and study outcomes. Through this approach, we aimed to identify potential diagnostic biomarkers associated with hemolytic processes in patients with AML and to elucidate novel pathophysiological mechanisms underlying hemolysis in this patient population.

## Materials and methods

### Data source

The dataset was obtained by transcriptome microarray analysis of human peripheral blood from patients with AML (*N* = 65) (M0:25; M1:11; M2:10; M3:1; M4:7; M4 eo t [16;16] ou inv [16]:4; M5:6; M6:1) and healthy human volunteers (healthy controls; *N* = 62).

## Identification of differentially expressed genes

First, we differentially analyzed data from peripheral blood samples in the GSE164191 (https://www.ncbi.nlm.nih.gov/geo/query/acc.cgi?acc=GSE164191) and GSE89565 (https://www.ncbi.nlm.nih.gov/geo/query/acc.cgi?acc=GSE89565) datasets using R software (version 4.3.1) and preprocessed them for batch correction and normalization. We then performed differentially expressed gene (DEG) analysis screening using the “limma” package. After analyzing the significance of expression level differences, the “pheatmap” and “ggplot2” R packages were used to generate volcano maps and DEG expression heat maps, respectively. By analyzing genome relationships and gene-phenotype correlations, WGCNA can identify gene lists with similar/synergistic interactions [18]. We constructed a gene co-expression network for AML using the “WGCNA” R package (version 1.72–1), and then evaluated the relevance of the different modules to the pathogenic mechanism of AML and identified the most relevant modules from WGCNA as the central genes.

## Screening of target genes and signaling pathway analysis

We first searched for related genes in the GeneCards database (www.genecards.org) using “hemolysis” as the keyword, and then took the intersection of these genes with the WGCNA-screened genes and DEGs to obtain intersecting genes [19]. These intersecting genes were regarded as candidate hub genes relevant to AML and the pathogenesis of hemolysis. Subsequently, we performed Gene Ontology (GO) and Kyoto Encyclopedia of Genes and Genomes (KEGG) enrichment analysis using the “clusterProfiler” R package to assist us in understanding the potential mechanisms of AML progression and pathogenesis [20]. The STRING database (https://string-db.org/) and Cytoscape software (https://cytoscape.org/) were used to construct protein–protein interaction (PPI) networks and search for hub genes.

## Nomogram model construction and immune cell analysis

We constructed a nomogram model to predict the risk of hemolysis in patients with AML using the “rms” package [21]. The performance of the nomogram model was evaluated by calculating Harrell’s concordance index, which assesses predictive power [22]. To validate the diagnostic effectiveness of the candidate biomarkers, we used the “ROC” package to construct a receiver operator characteristic (ROC) curve. The area under the ROC curve (AUC) was used as a measure of accuracy. The criterion 0.9 ≤ AUC < 1 was used to identify excellent accuracy. Using correlation analysis, we examined the relationship between the target genes and immune cells in patients with AML to investigate their roles in hemolysis.

## Mendelian randomization

All data analyzed in this study were obtained from a publicly available database. Two-sample MR was used to explore the causal association between hub genes and the risk of hemolysis in patients with AML with single nucleotide polymorphisms (SNPs) defined as instrumental variables. Hub gene data were obtained from a publicly available genome-wide association study (GWAS) data source. We selected two representative genes, *STAT1* and *TLR4*, and a leukemia inhibitory factor, as representatives of AML for MR analysis. Data for *STAT1* are available at https://gwas.mrcieu.ac.uk/datasets/?gwas_id__icontains=prot-a-2868&year__iexact=&trait__icontains=&consortium__icontains =, data on TLR4 is available at https://gwas.mrcieu.ac.uk/datasets/?gwas_id__icontains=prot-c-3647_49_4&year__iexact=&trait__icontains=&consortium__icontains =, and data for leukemia inhibitory factor are available at https://gwas.mrcieu.ac.uk/datasets/?gwas_id__icontains=prot-a-1736&year__iexact=&trait__icontains=&consortium__icontains =. MR analysis was performed using the “TwoSampleMR” package, and inverse variance weighting (IVW) was used to assess the relationship between hub gene levels and the risk of hemolysis in patients with AML. MR–Egger was used for additional sensitivity analyses [23].

## Results

### DEG screening

**Fig. 1:**
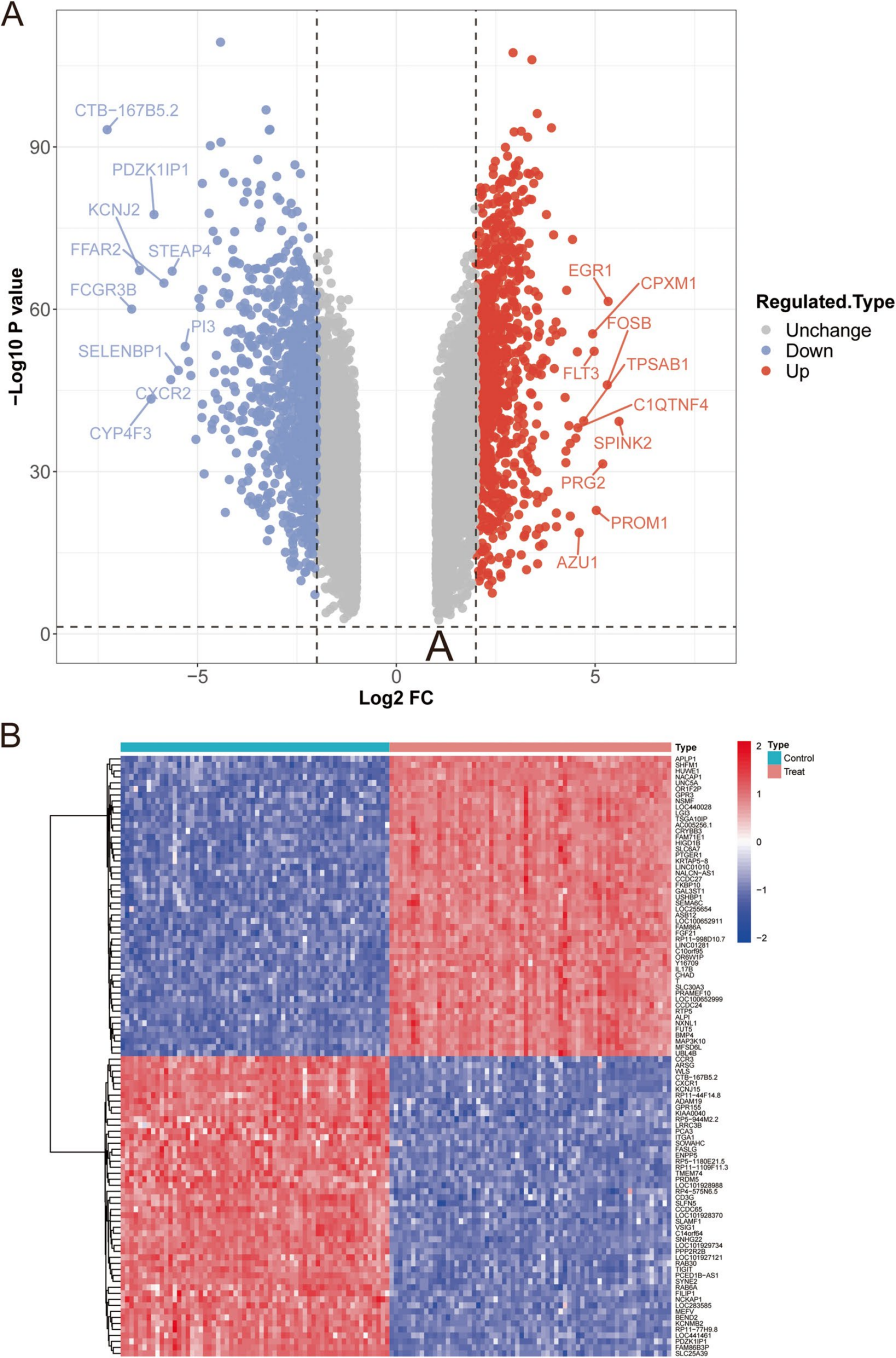
Genes differentially expressed between the AML patients and normal groups. **A** Volcanic map for differential expression analysis of GSE164191 and GSE89565. **B** Heat map for differential expression analysis of GSE164191 and GSE89565. Blue represents down-regulated genes, red represents up-regulated genes, and grey represents undifferentiated genes

We first used the Limma package in R software (V4.0.1) to perform differential analysis between peripheral blood data from patients AML (GSE89565) and healthy individuals (GSE164191) and found a total of 7,339 DEGs, of which 3,733 were upregulated and 3,606 were downregulated (Figs. 1A,​ B; Supplementary Table S1).

## Construction of WGCNA network and identification of an AML-related module

**Fig. 2:**
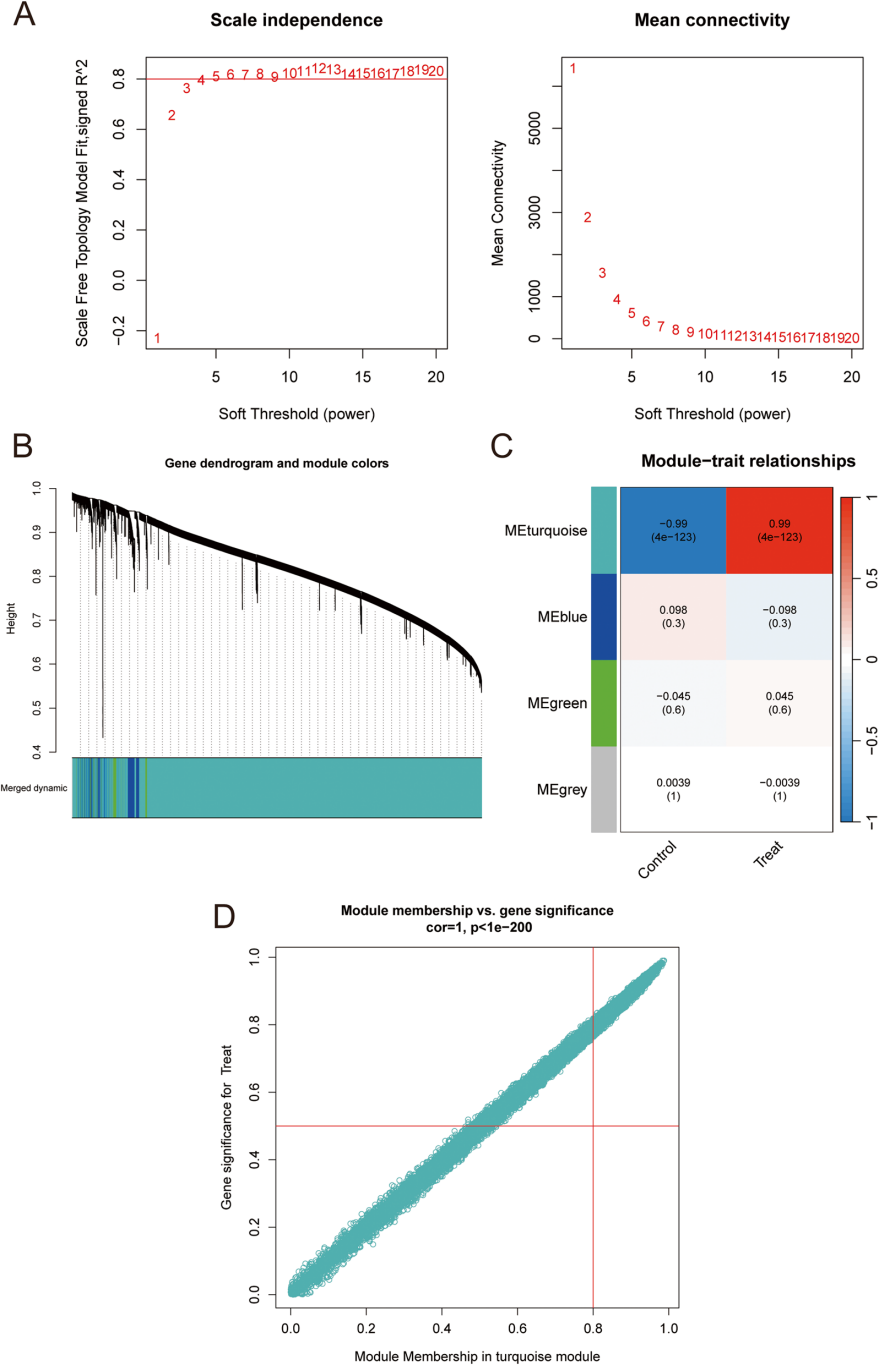
Identification of AML-associated gene modules in the GEO dataset using WGCNA. **A** The left panel shows scale independence and the right panel shows neighboring means to choose the best power value. **B** Dendrogram of all genes in GSE164191 and GSE89565 datasets were clustered on the basis of a topological overlap matrix (1-TOM). Each branch in the clustering tree represents a gene, while co-expression modules were constructed in different colors. **C** Module-trait heatmap of the correlation between the clustering gene module and AML in the dataset. Each module contains the corresponding correlation coefficient and *p* value. **D** Scatter plot of module turquoise has the strongest positive correlation with AML in the dataset

To identify potential gene modules that have a greater correlation with AML, we performed WGCNA on the peripheral blood datasets of patients with AML (GSE164191 and GSE89565) (Fig. 2A). By clustering the genes and patients separately, we identified four different modules, obtained positive correlation coefficients (Fig. 2B), and based on the correlations, the genes with significance greater than 0.5 and Module Membership greater than 0.8 in the turquoise module were selected for subsequent analysis (Fig. 2C and Supplementary Table S2).

## Parallel signaling pathway enrichment analysis of hemolysis-related genes in patients with AML

**Fig. 3:**
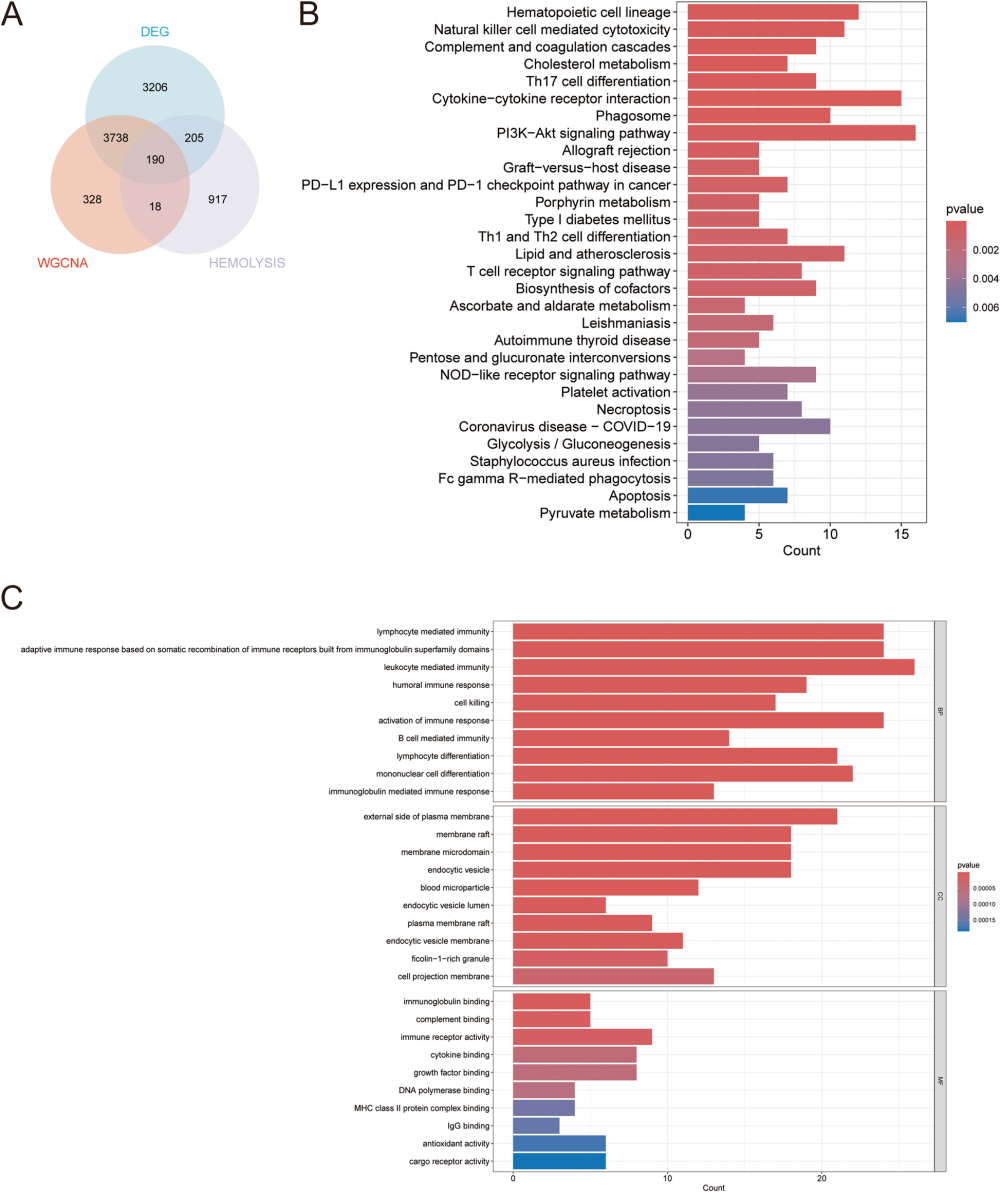
Candidate hub genes were screened and validated. **A** Venn diagram revealed 190 overlapping candidate hub genes. **B** GO enrichment analysis of candidate hub genes. **C** KEGG pathway analysis of candidate hub genes

We first searched the GeneCards database (www.genecards.org) for related genes using “hemolysis” as the keyword and obtained 1,330 genes (Supplementary Table S3). These genes, the hub genes in the WGCNA, and DEGs were screened using Venn diagrams, and 190 overlapping genes were identified as candidate hub genes that may play important roles in AML and hemolysis (Fig. 3A). We further explored the potential roles of these 190 overlapping genes using GO and KEGG analysis (Figs. 3B, C). GO enrichment analysis showed that the overlapping genes mainly affected lymphocyte-mediated immunity, leukocyte-mediated immunity, and the activation of immune responses. KEGG enrichment analysis showed that the overlapping genes mainly affected cytokine–cytokine receptor interactions, the hematopoietic cell lineage, and the PI3 K AKT pathway. These results revealed the important role of this group of genes in the immune response.

## PPI network analysis of the hub genes

**Fig. 4:**
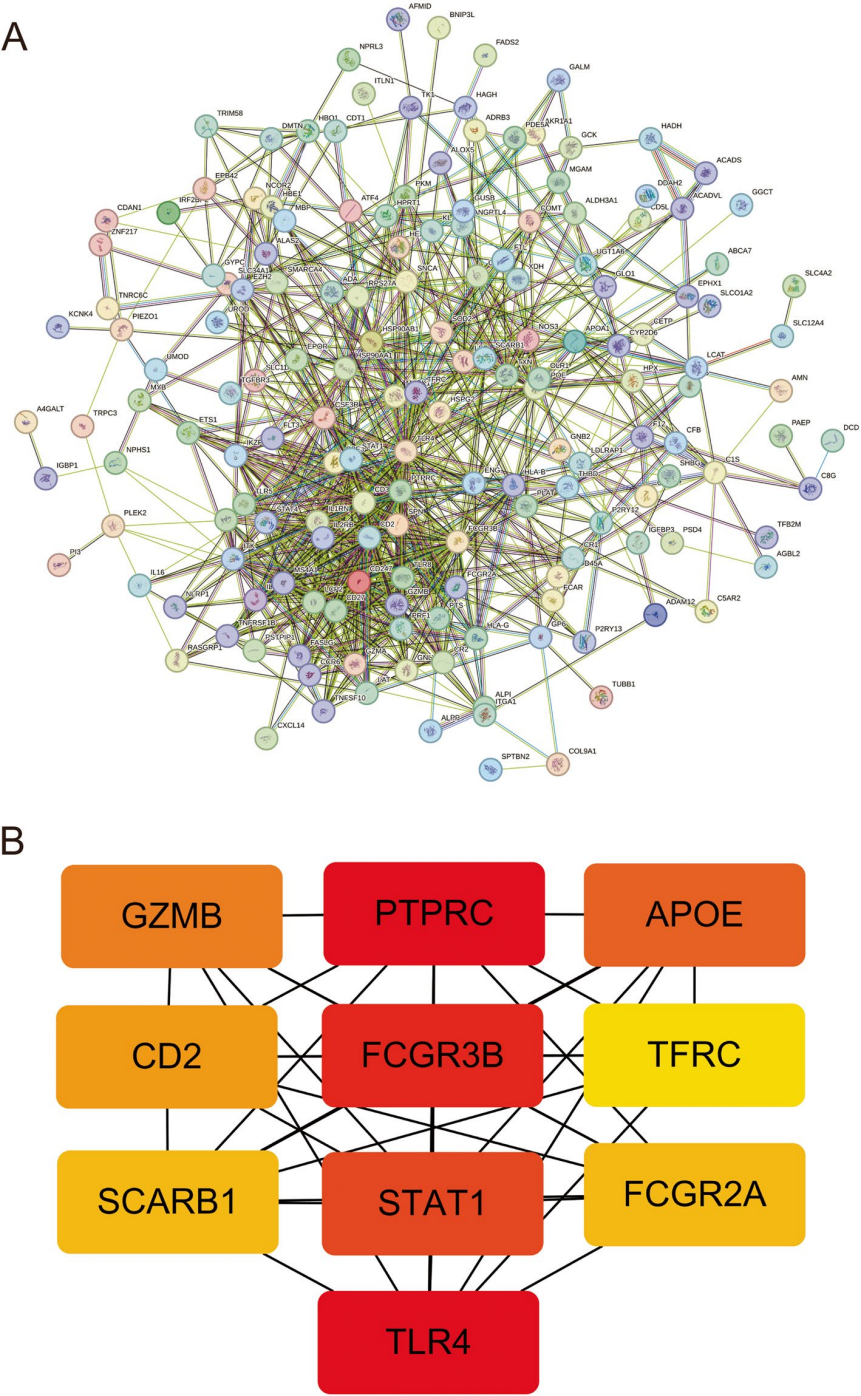
The construction of PPI network. **A** PPI network of overlapping hub genes. **B** The core genes of the interaction network were obtained by degree salgorithm

We used the STRING database to construct a PPI network of the overlapping hub genes (Fig. 4A). The presence of a line between the dots indicates an interaction between the two molecules, and different colors represent upregulation/downregulation. Subsequently, the top 10 highly ranked upregulated genes were visualized using Cytoscape software (Fig. 4B). Cells expressing PTPRC, FCGR3B, STAT1, TLR4, GZMB, CD2, SCARB1, APOE, TFRC, and FCGR2 A were sorted. A deeper color indicates a higher score.

## Construction of a nomogram model for predicting the risk of hemolysis in patients with AML

**Fig. 5:**
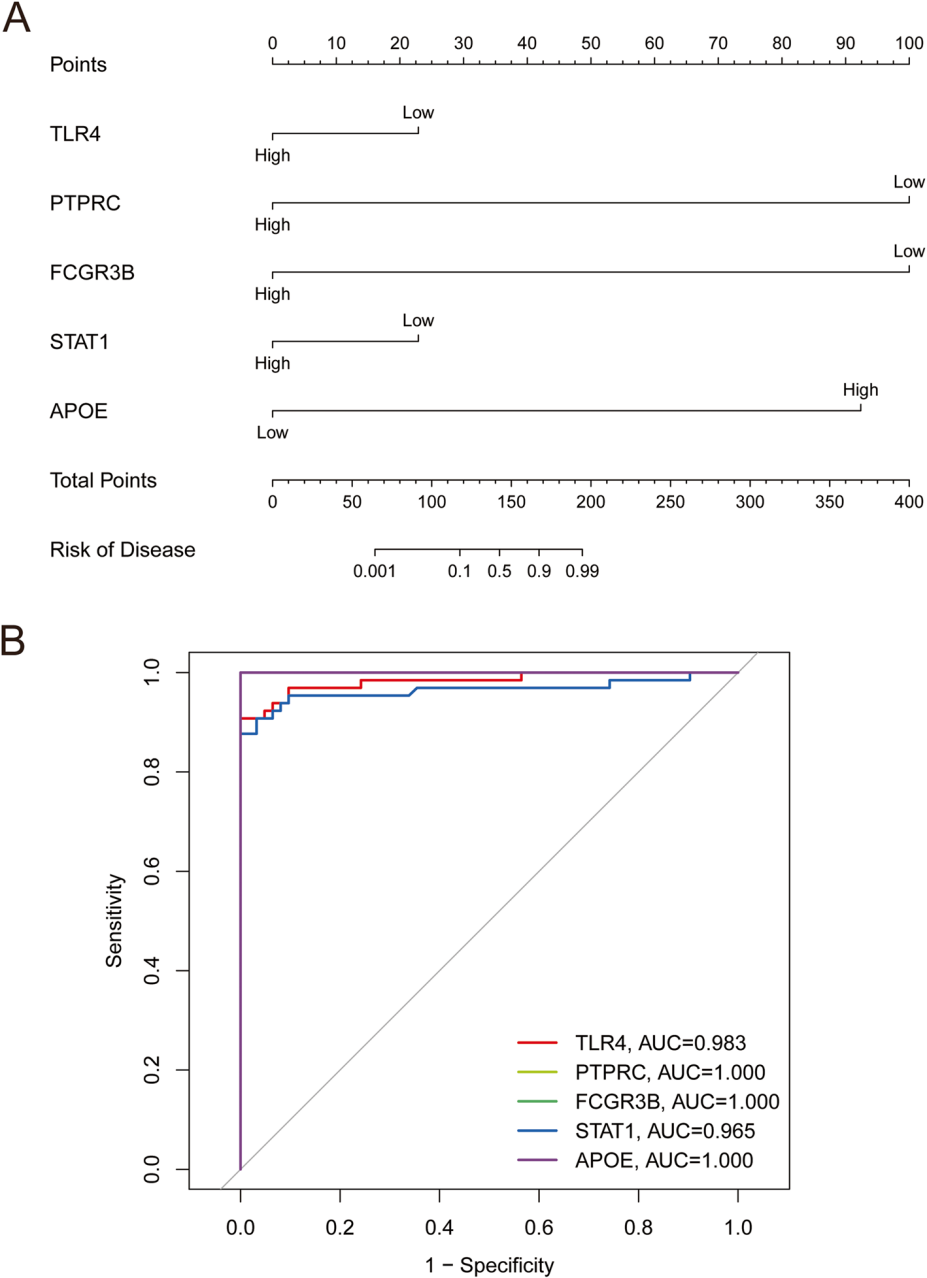
Predicting the risk of hemolysis in AML patients using nomograms. **A** Nomogram model of hub genes. **B** ROC curves to assess the diagnostic efficacy of nomogram model and each hub gene

We constructed a nomogram model to predict the risk of hemolysis in patients with AML (Fig. 5A). Our nomogram model performed well at predicting hemolysis in patients. Subsequently, we calculated the ROC curves for the five hub genes (*TLR4*, *PTPRC*, *FCGR3B*, *STAT1*, and *APOE*) to assess their diagnostic effect. The AUC of our nomogram differentiated between hemolysis in patients with AML and controls (Fig. 5B). The AUC values for *TLR4*, *PTPRC*, *FCGR3B*, *STAT1*, and *APOE* were 0.983, 1.000, 1.000, 0.965, and 1.000, respectively.

It is noteworthy that in our results, three hub genes, *PTPRC*, *FCGR3B*, and *APOE*, had AUC values of 1. APOE is a classical hemolysis inhibitory factor that protects erythrocytes from environmentally-induced lysis in a number of ways [24–26]. Meanwhile, in our PPI results, APOE had the lowest level of significance among the five genes. The expression of PTPRC, also known as CD45, gradually disappears during bone marrow erythropoiesis and is commonly used for subpopulation analysis, immunoassays of myeloid cells, and as a serum marker for some hemolytic diseases [27–29]. FCGR3B is a low-affinity immunoglobulin gamma Fc receptor III, also known as CD16, and a surface marker of immature erythrocytes. It can be used as a marker of nocturnal paroxysmal hemoglobinuria [30, 31] and can also indicate the occurrence of hematologic malignancy [32]. Therefore, we chose TLR4 for further analyses.

## Analysis of TLR4 correlations with immune cells

**Fig. 6:**
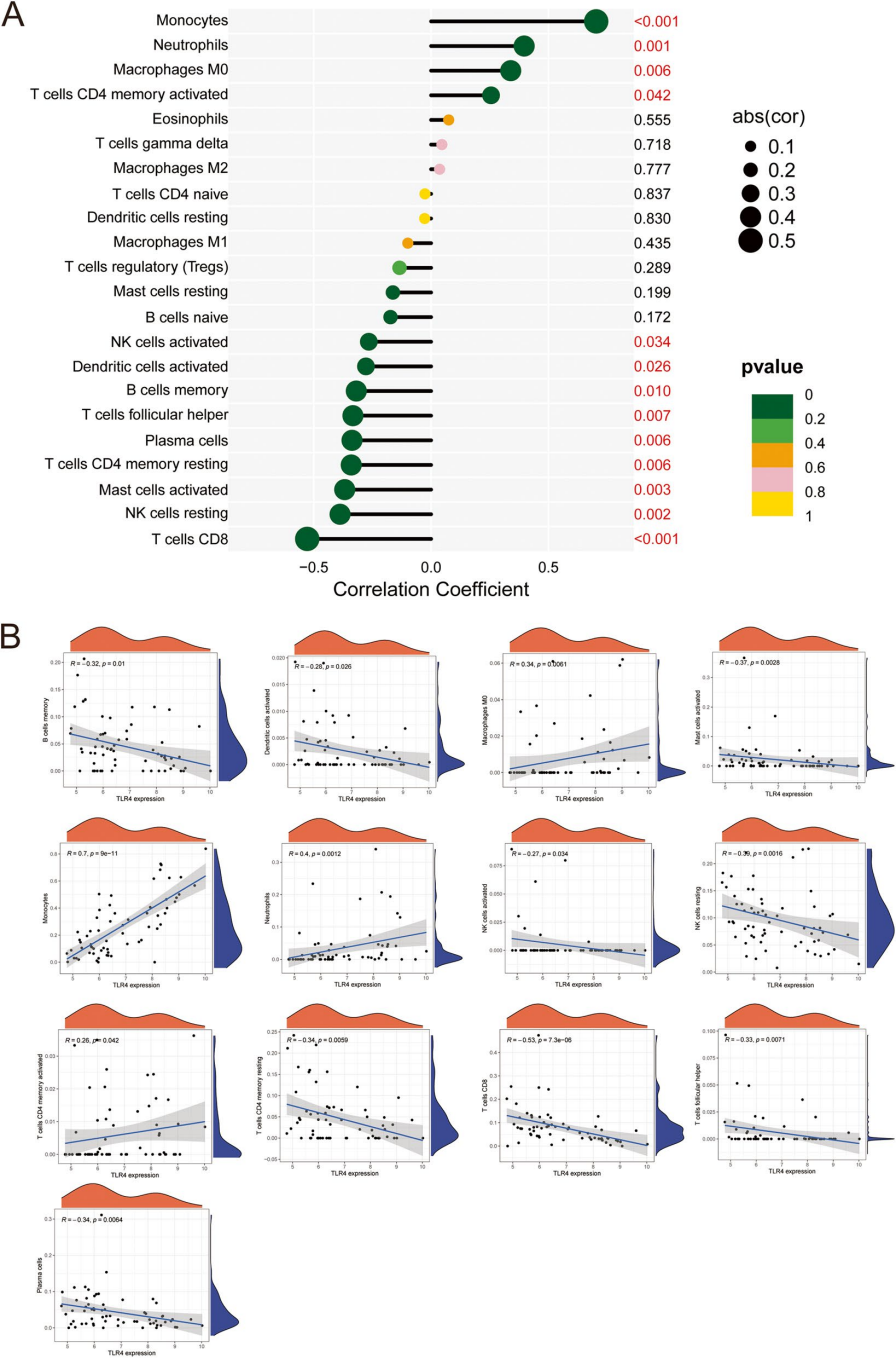
Correlation of TLR4 with immune cells. **A** A general overview of the relationship between TLR4 and immune cells, the numbers on the right are p-values and those that are statistically significant have been marked in red. **B** Scatter plots of TLR4 expression respectively and immune cell expression, only immune cells with *P* < 0.05 are listed

KEGG pathway analysis and GO enrichment showed that these hub genes were primarily associated with cytokine–cytokine receptor interactions. A positive correlation was observed between monocytes, neutrophils, macrophages (M0), CD4 + memory-activated T cells, and TLR4 expression levels, whereas activated natural killer (NK) cells, dendritic cells, memory B cells, helper T cells, plasma cells, resting memory T cells, resting NK cells, CD8-positive T cells, and TLR4 expression levels were negatively correlated (Figs. 6A, B). These results demonstrated a direct relationship between immune cells and the hub gene *TLR4* in AML.

## TLR4 is causally associated with the risk of hemolysis in patients with AML

**Fig. 7:**
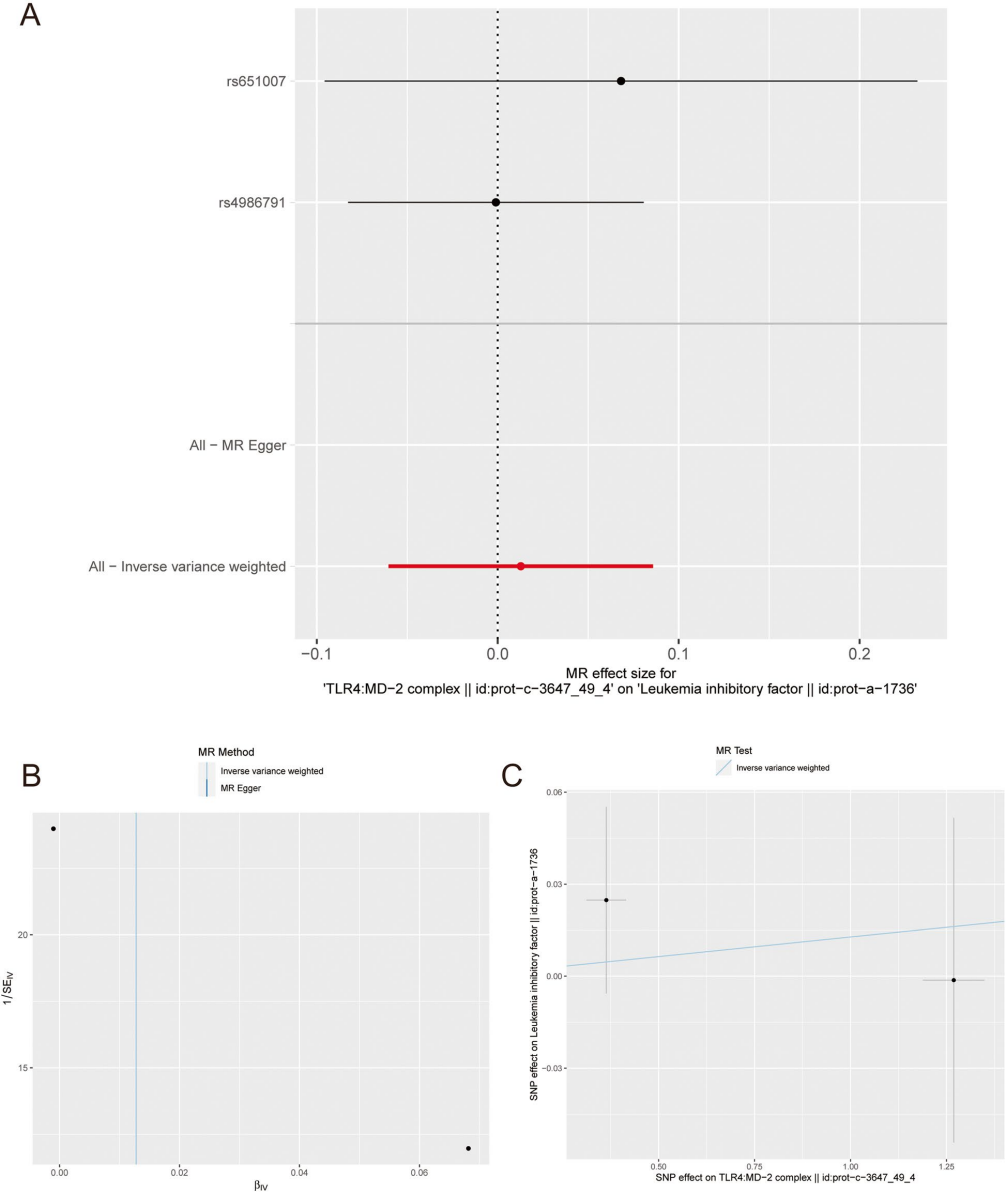
Mendelian randomization study results. **A** Scatter plot showing the causal effect of TLR4 on the risk of hemolysis in AML patients. **B** Forest plot showing the causal effect of each SNP on the risk of hemolysis in AML patients. **C** Funnel plots to visualize overall heterogeneity of MR estimates for the effect of TLR4 on the risk of hemolysis in AML patients

The SNP characteristics of *TLR4* and the risk of hemolysis in patients with AML are shown in Supplementary Table S4. No SNP was a weak instrumental variable. Figures 7A and B present the causal effects of each genetic variant on the risk of hemolysis in patients with AML. We assessed the causal association between TLR4 levels and risk of hemolysis in patients with AML. Using the IVW method, we found that TLR4 levels were associated with the risk of hemolysis in patients with AML, with an odds ratio of 1.013 (95% confidence interval = 1.001–1.045, *p* = 0.031216). No horizontal pleiotropy was observed at the intercept of the MR-Egger regression (*p* = 0.376), indicating that pleiotropy did not influence the causal effect of the funnel plot (Fig. 7C). Despite these consistent results, all SNPs were found to be significant for causality. Patients with AML with high TLR4 levels and hemolysis risk did not have a dominant SNP, and the previous MR results were valid.

## Discussion

AML is a hematological malignancy characterized by hemorrhage, fatigue, infection, and the failure of myeloblasts or pregranulocytes to differentiate normally [2, 33]. Hemolysis occurs in patients with AML for various reasons, including thrombocytopenia [34], TAM [11], hemolysis after bone marrow transplantation [12], and therapy-related hemolysis [13]. Hemolytic manifestations are common in pediatric patients (≤ 15 years of age) at first diagnosis, mostly manifesting as hepatosplenomegaly and abnormal laboratory tests [35]. In adults, hemolysis most often manifests as post-treatment hemolysis [36], which is one of the criteria for evaluating treatment efficacy and the patient’s prognosis [37, 38]. Classical cytotoxic treatment of AML (a combination of 7 days of cytarabine and 3 days of anthracycline) also leads to hemolysis, thrombocytopenic hemorrhage, and granulocytopenia, which often imply a poor prognosis [39, 40]. Here, we explored the specific biological mechanisms that produce hemolysis in patients AML using bioinformatics methods, clarified the causal relationship between hemolysis and hub genes in patients with AML, and provided new perspectives on the specific course of AML treatment and progression.

We first utilized data from the Gene Expression Omnibus database for differential analysis, identified DEGs, and then intersected them with hub genes in WGCNA and hemolysis-related genes in the GeneCards database to obtain marker genes for hemolysis in patients with AML. We then used PPI network analysis and Cytoscape software to screen hub genes, and obtained 10 hub genes. Five genes, *TLR4*, *PTPRC*, *FCGR3B*, *STAT1*, and *APOE* that were significantly associated with patient prognosis were identified by establishing a nomogram model of prognostic risk.

PTPRC (CD45) and FCGR3B (CD16) are expressed in immature erythrocytes and leukocytes (T cells, NK cells, and B cells) and are commonly used for blood cell subpopulation analysis and immune status analysis [27, 28]. Consistent with our findings, both CD45 and CD16 are markers of certain hemolytic disorders (paroxysmal sleep hemoglobinuria) [29–31], and CD16 is a marker of certain hematologic neoplasms (myelodysplastic syndromes) [32]. However, the roles of CD45 and CD16 in the development of hemolysis are not clear. The role of STAT1 in AML is also controversial. Nitulescu et al. experimentally demonstrated that STAT1 activates the JAK-STAT pathway through phosphorylation at Ser727 to promote AML growth and malignant biological behaviors [41], whereas Gao et al. mediated the phosphorylation of the 727 site using diptoindonesin G to promote AML cell differentiation through the ERK pathway [42]. However, similar to CD45 and CD16, the role of STAT1 in hemolysis in patients is unclear.

Apolipoprotein E (APOE) is involved in the conversion and metabolism of lipoproteins and regulates many biological functions. APOE/TLR4 values have been reported to be critical for lipid metabolism [43]. By downloading AML-related data from The Cancer Genome Atlas database and performing differential analysis using edgeR, Huang et al. found APOE to be the hub gene in patients with a mutation of *TP53*, which is the driving gene of AML [44]. However, they used only the R package edgeR for differential analysis and did not validate or evaluate the effectiveness of the candidate biomarkers for diagnostic use through mathematical modeling. More robust and comprehensive designs should be considered when using these bioinformatic approaches to study diseases.

TLR4 is a pattern-recognizing receptor [45]. It recognizes intracellular and extracellular ligands, mediates inflammatory suppression, and maintains an environment conducive to AML cells [45]. In an in vitro study, Hu et al. found that TLR4 regulates the inflammatory response of macrophages through the AP- 1 signaling pathway in AML [46]. Moreover, Baakhlagh et al. found that AML cell proliferation decreases after inhibiting TLR4 activity [47]. In addition, Chakraborty et al. reported that the TLR4/NLR signaling pathway promotes ineffective hematopoiesis through the activation of damage-associated molecular patterns, which contribute to the development of AML [48]. We found that TLR4 was involved in the development of AML and in the occurrence of hemolysis in patients with AML; thus, it is clear that TLR4 still has broad research prospects in the field of AML.

This is the first study to explore the causal association between TLR4 levels and AML risk using a two-sample MR analysis based on a large amount of GWAS data on TLR4 (exposure) and AML (outcome). The findings of this MR study suggest that serum TLR4 levels are causally associated with an increased risk of hemolysis in patients with AML.

Despite the significance of our study, it has some shortcomings. First, only one AML dataset was used, because it was difficult to find a healthy control population cohort from a relevant database. These results would have been more convincing if more AML datasets from different populations were combined. Second, this study only used bioinformatics to analyze hub genes and their potential functions associated with the occurrence of hemolysis in patients with AML, and more biological experiments are needed to confirm the specific mechanisms of the identified hub genes.

In conclusion, we constructed a WGCNA-based co-expression network and identified hemolysis-associated AML genes. This may help in the development of pre-symptomatic diagnostics and provide further insights into the molecular mechanisms underlying hemolysis-associated AML risk genes.

## Supplementary Information

Supplementary Material 1.

Supplementary Material 2.

Supplementary Material 3.

Supplementary Material 4.

Supplementary Material 5.

## Data Availability

This research used GSE89565 and GSE164191. We collect the GSE89565 and GSE164191 datasets from the NCBI Gene Expression Omnibus (GEO) database (https://www.ncbi.nlm.nih.gov/geo/). All data and code used in this paper can be obtained by contacting the corresponding author.

## References

[1] Siegel RL, Miller KD, Jemal A (2019) Cancer statistics, 2019. CA: A Cancer Journal for Clinicians 69:7–34. 10.3322/caac.21551. 10.3322/caac.21551 30620402

[2] De Kouchkovsky I, Abdul-Hay M. AML: a comprehensive review and 2016 update. Blood Cancer J. 2016;6:e441–e441. 10.1038/bcj.2016.50. 27367478 10.1038/bcj.2016.50 PMC5030376

[3] Siegel RL, Miller KD, Jemal A (2017) Cancer statistics, 2017. CA: A Cancer Journal for Clinicians 67:7–30. 10.3322/caac.21387. 10.3322/caac.21387 28055103

[4] Siegel RL, Miller KD, Wagle NS, Jemal A (2023) Cancer statistics, 2023. CA: A Cancer Journal for Clinicians 73:17–48. 10.3322/caac.21763 . 10.3322/caac.21763 36633525

[5] Siegel RL, Giaquinto AN, Jemal A (2024) Cancer statistics, 2024. CA: A Cancer Journal for Clinicians 74:12–49. 10.3322/caac.21820 . 10.3322/caac.21820 38230766

[6] Leung WK, Torres Chavez AG, French-Kim M, et al (2024) Targeting IDH2R140Q and other neoantigens in AML. Blood Journal. 2023021979. 10.1182/blood.2023021979. 10.1182/blood.2023021979 PMC11103096 38241630

[7] Pollyea DA, Bixby D, Perl A, et al. NCCN Guidelines Insights: AML, Version 2.2021: featured updates to the NCCN Guidelines. J Natl Compr Canc Netw. 2021;19:16–27. 10.6004/jnccn.2021.0002. 33406488 10.6004/jnccn.2021.0002

[8] Sekeres MA, Guyatt G, Abel G, et al. American Society of Hematology 2020 guidelines for treating newly diagnosed AML in older adults. Blood Adv. 2020;4:3528–49. 10.1182/bloodadvances.2020001920. 32761235 10.1182/bloodadvances.2020001920 PMC7422124

[9] Jaime-Pérez JC, García-Salas G, Áncer-Rodríguez J, Gómez-Almaguer D. Audit of red blood cell transfusion in patients with acute leukemia at a tertiary care university hospital. Transfusion. 2020;60:724–30. 10.1111/trf.15700. 32056229 10.1111/trf.15700

[10] Ballo O, Fleckenstein P, Eladly F, et al. Reducing the red blood cell transfusion threshold from 8·0 g/dl to 7·0 g/dl in acute myeloid leukaemia patients undergoing induction chemotherapy reduces transfusion rates without adversely affecting patient outcome. Vox Sang. 2020;115:570–8. 10.1111/vox.12919. 32342521 10.1111/vox.12919

[11] Regierer AC, Kuehnhardt D, Schulz C-O, et al. Breast cancer-associated thrombotic microangiopathy. Breast Care. 2011;6:441–5. 10.1159/000335201. 22419897 10.1159/000335201 PMC3290020

[12] Hamamyh T, Yassin MA. Autoimmune hemolytic anemia in chronic myeloid leukemia. Pharmacology. 2020;105:630–8. 10.1159/000507295. 32485715 10.1159/000507295 PMC7845422

[13] Tesfazghi MT, Farnsworth CW, Roper SM, et al. Confounding case of hemolysis in a patient with acute leukemia. Clin Chem. 2018;64:1690–4. 10.1373/clinchem.2017.284042. 30487187 10.1373/clinchem.2017.284042

[14] Gamis AS, Howells WB, DeSwarte-Wallace J, et al. Alpha hemolytic streptococcal infection during intensive treatment for AML: a report from the Children’s cancer group study CCG-2891. J Clin Oncol. 2000;18:1845–55. 10.1200/JCO.2000.18.9.1845. 10784625 10.1200/JCO.2000.18.9.1845

[15] Tan K-B, Ling L-U, Bunte RM, et al. In vivo efficacy of a novel liposomal formulation of safingol in the treatment of AML. J Control Release. 2012;160:290–8. 10.1016/j.jconrel.2011.11.002. 22100388 10.1016/j.jconrel.2011.11.002

[16] Tan K-B, Ling L-U, Bunte RM, et al. Liposomal codelivery of a synergistic combination of bioactive lipids in the treatment of AML. Nanomedicine (Lond). 2014;9:1665–79. 10.2217/nnm.13.123. 24294981 10.2217/nnm.13.123

[17] Kang L, Han X, Chang X, et al. Redox-sensitive self-assembling polymer micelles based on oleanolic modified hydroxyethyl starch: Synthesis, characterisation, and oleanolic release. Int J Biol Macromol. 2024;266: 131211. 10.1016/j.ijbiomac.2024.131211. 38552688 10.1016/j.ijbiomac.2024.131211

[18] Langfelder P, Horvath S. WGCNA: an R package for weighted correlation network analysis. BMC Bioinformatics. 2008;9:559. 10.1186/1471-2105-9-559. 19114008 10.1186/1471-2105-9-559 PMC2631488

[19] Safran M, Rosen N, Twik M, et al (2021) The GeneCards Suite. In: Abugessaisa I, Kasukawa T (eds) Practical Guide to Life Science Databases. Springer Nature Singapore, Singapore, pp 27–56.

[20] Wu T, Hu E, Xu S, et al (2021) clusterProfiler 4.0: A universal enrichment tool for interpreting omics data. The Innovation 2:100141. 10.1016/j.xinn.2021.100141. 10.1016/j.xinn.2021.100141 PMC8454663 34557778

[21] Iasonos A, Schrag D, Raj GV, Panageas KS. How to build and interpret a nomogram for cancer prognosis. J Clin Oncol. 2008;26:1364–70. 10.1200/JCO.2007.12.9791. 18323559 10.1200/JCO.2007.12.9791

[22] Harrell FE, Lee KL, Mark DB. Multivariable prognostic models: issues in developing models, evaluating assumptions and adequacy, and measuring and reducing errors. Stat Med. 1996;15:361–87. 10.1002/(SICI)1097-0258(19960229)15:4%3c361::AID-SIM168%3e3.0.CO;2-4. 8668867 10.1002/(SICI)1097-0258(19960229)15:4<361::AID-SIM168>3.0.CO;2-4

[23] Dudbridge F. Polygenic Mendelian randomization. Cold Spring Harb Perspect Med. 2021;11: a039586. 10.1101/cshperspect.a039586. 32229610 10.1101/cshperspect.a039586 PMC7849343

[24] Vogt LM, Kwasniewicz E, Talens S, et al. Apolipoprotein E triggers complement activation in joint synovial fluid of rheumatoid arthritis patients by binding C1q. J Immunol. 2020;204:2779–90. 10.4049/jimmunol.1900372. 32253242 10.4049/jimmunol.1900372 PMC7313146

[25] Giunta S, Galeazzi R, Valli MB, et al. Transferrin neutralization of amyloid beta 25–35 cytotoxicity. Clin Chim Acta. 2004;350:129–36. 10.1016/j.cccn.2004.07.025. 15530469 10.1016/j.cccn.2004.07.025

[26] Galeazzi L, Corder EH, Galeazz R, et al. In vitro apolipoprotein E protects human red blood cells against lysis induced by amyloid-beta (AP) fragment 25–35. Amyloid. 2002;9:103–7. 12440482

[27] Barcellini W, Zaninoni A, Imperiali FG, et al. Anti-erythroblast autoimmunity in early myelodysplastic syndromes. Haematologica. 2007;92:19–26. 10.3324/haematol.10546. 17229631 10.3324/haematol.10546

[28] Boulais PE, Mizoguchi T, Zimmerman S, et al. The majority of CD45- Ter119- CD31- bone marrow cell fraction is of hematopoietic origin and contains erythroid and lymphoid progenitors. Immunity. 2018;49:627-639.e6. 10.1016/j.immuni.2018.08.019. 30314756 10.1016/j.immuni.2018.08.019 PMC6377266

[29] Wong SA, Dalal BI, Leitch HA. Paroxysmal nocturnal hemoglobinuria testing in patients with myelodysplastic syndrome in clinical practice-frequency and indications. Curr Oncol. 2018;25:e391–7. 10.3747/co.25.4018. 30464689 10.3747/co.25.4018 PMC6209566

[30] Kotru M, Sharma R, Pramanik SK, et al. Value of CD16/CD66b/CD45 in comparison to CD55/CD59/CD45 in diagnosis of paroxysmal nocturnal haemoglobinuria: An Indian experience. Indian J Med Res. 2017;146:362–8. 10.4103/ijmr.IJMR_195_14. 29355143 10.4103/ijmr.IJMR_195_14 PMC5793471

[31] Höchsmann B, Rojewski M, Schrezenmeier H. Paroxysmal nocturnal hemoglobinuria (PNH): higher sensitivity and validity in diagnosis and serial monitoring by flow cytometric analysis of reticulocytes. Ann Hematol. 2011;90:887–99. 10.1007/s00277-011-1177-4. 21359652 10.1007/s00277-011-1177-4 PMC3132386

[32] Wang SA, Pozdnyakova O, Jorgensen JL, et al. Detection of paroxysmal nocturnal hemoglobinuria clones in patients with myelodysplastic syndromes and related bone marrow diseases, with emphasis on diagnostic pitfalls and caveats. Haematologica. 2009;94:29–37. 10.3324/haematol.13601. 19001281 10.3324/haematol.13601 PMC2625410

[33] Geetha N, Sreelesh KP, Priya MJ, et al. Osteolytic Bone lesions - A rare presentation of AML M6. Mediterrean Journal of Hematology and Infectious Diseases. 2015;7: e2015017. 10.4084/MJHID.2015.017. 10.4084/MJHID.2015.017 PMC4344168 25745544

[34] Sandhow L, Cai H, Leonard E, et al. Skin mesenchymal niches maintain and protect AML-initiating stem cells. J Exp Med. 2023;220: e20220953. 10.1084/jem.20220953. 37516911 10.1084/jem.20220953 PMC10373345

[35] Seth R, Singh A. Leukemias in children. Indian J Pediatr. 2015;82:817–24. 10.1007/s12098-015-1695-5. 25680783 10.1007/s12098-015-1695-5

[36] Döhner H, Estey E, Grimwade D, et al. Diagnosis and management of AML in adults: 2017 ELN recommendations from an international expert panel. Blood. 2017;129:424–47. 10.1182/blood-2016-08-733196. 27895058 10.1182/blood-2016-08-733196 PMC5291965

[37] Gupta S, Baxter NN, Sutradhar R, et al. Adolescents and young adult AML outcomes at pediatric versus adult centers: A population-based study. Pediatric Blood Cancer. 2021;68: e28939. 10.1002/pbc.28939. 33559361 10.1002/pbc.28939

[38] Truong TH, Pole JD, Barber R, et al. Enrollment on clinical trials does not improve survival for children with AML: A population-based study. Cancer. 2018;124:4098–106. 10.1002/cncr.31728. 30291800 10.1002/cncr.31728

[39] Schmitt A, Li L, Giannopoulos K, et al. Quantitative expression of Toll-like receptor-2, -4, and -9 in dendritic cells generated from blasts of patients with AML. Transfusion. 2008;48:861–70. 10.1111/j.1537-2995.2007.01616.x. 18208411 10.1111/j.1537-2995.2007.01616.x

[40] Döhner H, Weisdorf DJ, Bloomfield CD. AML. N Engl J Med. 2015;373:1136–52. 10.1056/NEJMra1406184. 26376137 10.1056/NEJMra1406184

[41] Nitulescu II, Meyer SC, Wen QJ, et al. Mediator kinase phosphorylation of STAT1 S727 promotes growth of neoplasms with JAK-STAT activation. EBioMedicine. 2017;26:112–25. 10.1016/j.ebiom.2017.11.013. 29239838 10.1016/j.ebiom.2017.11.013 PMC5832629

[42] Gao J, Fan M, Xiang G, et al. Diptoindonesin G promotes ERK-mediated nuclear translocation of p-STAT1 (Ser727) and cell differentiation in AML cells. Cell Death Dis. 2017;8: e2765. 10.1038/cddis.2017.159. 28471454 10.1038/cddis.2017.159 PMC5520695

[43] Sorrentino R, Yilmaz A, Schubert K, et al. A single infection with *Chlamydia pneumoniae* is sufficient to exacerbate atherosclerosis in ApoE deficient mice. Cell Immunol. 2015;294:25–32. 10.1016/j.cellimm.2015.01.007. 25666507 10.1016/j.cellimm.2015.01.007 PMC4391498

[44] Huang R, Liao X, Li Q. Identification of key pathways and genes in TP53 mutation AML: evidence from bioinformatics analysis. Onco Targets Ther. 2018;11:163–73. 10.2147/OTT.S156003. 29343974 10.2147/OTT.S156003 PMC5749383

[45] Bruserud Ø, Reikvam H, Brenner AK. Toll-like receptor 4, osteoblasts and eukemogenesis; the lesson from AML. Molecules. 2022;27:735. 10.3390/molecules27030735. 35163998 10.3390/molecules27030735 PMC8838156

[46] Hu X, Zhou J, Song S-S, et al. TLR4/AP-1-targeted anti-inflammatory intervention attenuates insulin sensitivity and liver steatosis. Mediators Inflamm. 2020;2020:2960517. 10.1155/2020/2960517. 33013197 10.1155/2020/2960517 PMC7519185

[47] Baakhlagh S, Kashani B, Zandi Z, et al. Toll-like receptor 4 signaling pathway is correlated with pathophysiological characteristics of AML patients and its inhibition using TAK-242 suppresses AML cell proliferation. Int Immunopharmacol. 2021;90: 107202. 10.1016/j.intimp.2020.107202. 33278749 10.1016/j.intimp.2020.107202

[48] Chakraborty S, Shapiro LC, de Oliveira S, et al. Therapeutic targeting of the inflammasome in myeloid malignancies. Blood Cancer J. 2021;11:152. 10.1038/s41408-021-00547-8. 34521810 10.1038/s41408-021-00547-8 PMC8440507

