# Supplementary material for: Analysis of hemolysis-associated acute myeloid leukemia genes obtained using weighted gene co-expression network analysis and a Mendelian randomization study
Source: Blood Res. 2025 Apr 11;60(1):24. doi: 10.1007/s44313-025-00073-7 (PMC11992295; doi:10.1007/s44313-025-00073-7)
Supplement: Supplementary file 5 — Supplementary Material 5. [file 44313_2025_73_MOESM5_ESM.docx]

Supplementary Table S1. Genes differentially expressed between the AML patients and normal groups.

Supplementary Table S2. The turquoise model of hub genes.

Supplementary Table S3. Hemolysis-related genes from the Gene-Cards database.

Supplementary Table S4. The SNP characteristics of TLR4 and the risk of hemolysis in AML patients.
